# 2-[2-(1,3-Dioxoisoindolin-2-yl)acetamido]­acetic acid

**DOI:** 10.1107/S1600536810043047

**Published:** 2010-10-30

**Authors:** Moazzam H. Bhatti, Uzma Yunus, Imtiaz-ud-Din, S. Shams-ul-Islam, Wai-Yeung Wong

**Affiliations:** a Department of Chemistry, Allama Iqbal Open University, Islamabad, Pakistan; b Department of Chemistry, Hong Kong Baptist University, Waterloo Road, Kowloon Tong, Hong Kong

## Abstract

The title mol­ecule, C_12_H_10_N_2_O_5_, is non-planar with dihedral angles of 89.08 (7) and 83.21 (7)° between the phthalimide and acetamide mean planes, and the acetamide and acetic acid mean planes, respectively. In the crystal, symmetry-related mol­ecules are linked *via* N—H⋯O and O—H⋯O hydrogen bonds, forming an undulating two-dimensional network. There are also a number of weak C—H⋯O inter­actions, leading to the formation of a three-dimensional arrangement.

## Related literature

For the structures and biological properties of phthalimides and various derivatives, see: Antunes *et al.* (1998 ▶); Barooah & Baruah (2007 ▶); Barooah *et al.* (2006 ▶); Khan *et al.* (2002 ▶); Sharma *et al.* (2010 ▶); Yunus *et al.* (2008 ▶). For standard bond lengths, see: Allen *et al.* (1987 ▶). For bond lengths and angles in the phthalimide group, see: Feeder & Jones (1996 ▶); Ng (1992 ▶).
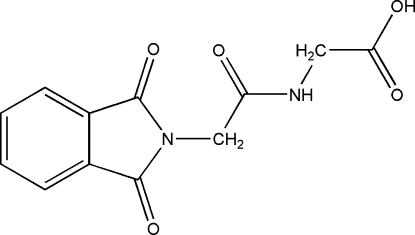


## Experimental

### 

#### Crystal data

C_12_H_10_N_2_O_5_
*M* *_r_* = 262.22
Monoclinic, 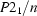

*a* = 4.8195 (5) Å
*b* = 10.3415 (11) Å
*c* = 22.629 (2) Å
β = 90.17 (1)°
*V* = 1127.9 (2) Å^3^
*Z* = 4
Mo *K*α radiation
μ = 0.12 mm^−1^
*T* = 173 K
0.34 × 0.24 × 0.20 mm

#### Data collection

Bruker SMART CCD diffractometer
Absorption correction: multi-scan (*SADABS*; Sheldrick, 1996 ▶) *T*_min_ = 0.939, *T*_max_ = 1.000
6788 measured reflections
2731 independent reflections
2533 reflections with *I* > 2σ(*I*)
*R*_int_ = 0.015

#### Refinement

*R*[*F* ^2^ > 2σ(*F* ^2^)] = 0.037
*wR*(*F* ^2^) = 0.094
*S* = 1.07
2731 reflections
180 parameters
H atoms treated by a mixture of independent and constrained refinement
Δρ_max_ = 0.33 e Å^−3^
Δρ_min_ = −0.24 e Å^−3^

### 

Data collection: *SMART* (Bruker, 2007 ▶); cell refinement: *SAINT* (Bruker, 2007 ▶); data reduction: *SAINT*; program(s) used to solve structure: *SHELXS97* (Sheldrick, 2008 ▶); program(s) used to refine structure: *SHELXL97* (Sheldrick, 2008 ▶); molecular graphics: *SHELXTL* (Sheldrick, 2008 ▶) and *Mercury* (Macrae *et al.*, 2006 ▶); software used to prepare material for publication: *SHELXTL*.

## Supplementary Material

Crystal structure: contains datablocks I, global. DOI: 10.1107/S1600536810043047/su2220sup1.cif

Structure factors: contains datablocks I. DOI: 10.1107/S1600536810043047/su2220Isup2.hkl

Additional supplementary materials:  crystallographic information; 3D view; checkCIF report

## Figures and Tables

**Table 1:** Hydrogen-bond geometry (Å, °)

*D*—H⋯*A*	*D*—H	H⋯*A*	*D*⋯*A*	*D*—H⋯*A*
N2—H2⋯O1^i^	0.908 (18)	2.172 (19)	3.0208 (13)	155.3 (17)
O5—H5⋯O3^ii^	0.93 (2)	1.67 (2)	2.5777 (13)	165.4 (17)
C2—H2*A*⋯O5^iii^	0.95	2.52	3.3142 (17)	141
C9—H9*A*⋯O4^iv^	0.99	2.56	3.2407 (15)	126
C9—H9*B*⋯O4^v^	0.99	2.59	3.3378 (15)	132
C11—H11*A*⋯O5^i^	0.99	2.48	3.4364 (14)	162

Symmetry codes: (i) 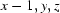; (ii) 
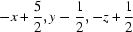
; (iii) 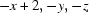; (iv) 
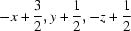
; (v) 
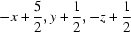
.

**Fig. 1.**
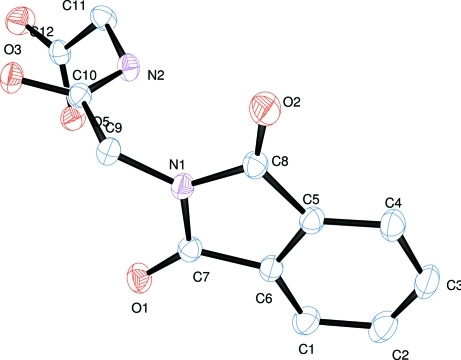
A view of the molecular structure of the title molecule, with displacement ellipsodes drawn at the 50% probability level.

**Fig. 2.**
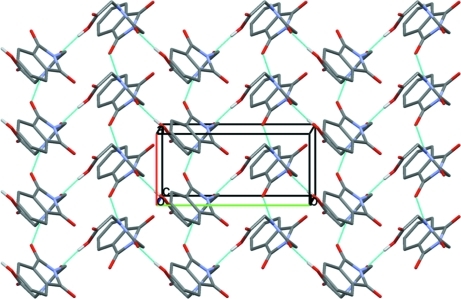
The crystal packing viewed along the c axis of the title compound, showing the N-H···O and O-H···O hydrogen bonds as cyan lines (H-atoms not involved in hydrogen bonding have been omitted for clarity).

## supplementary crystallographic information

### Comment

Phthalimides and its derivatives are one of the important class of organic molecules that possess diverse structural (Barooah & Baruah, 2007) and biological applications (Sharma *et al.*, 2010). Among phthalimides derivatives, N-phthaloylglycine has been the most widely studied for its metal complexes with supramolecular structures (Barooah *et al.*, 2006), kinetic studies for cleavage with various amines (Khan & Ismail, 2002) and heterocyclic derivatives such as oxadiazole (Antunes *et al.*, 1998) and 1,2,4-triazole (Yunus *et al.*, 2008). In the present investigation we report on the crystal structure of an acetamide derivative of the N-phthaloylglycine moiety.

The molecular structure of the title molecule is illustrated in Fig. 1. As a whole the molecule is non-planar and consists of three groups, namely phthalimide, acetamide and acetic acid, which are individually planar. The dihedral angle between the phthalimide (N1/C8/C5/C6/C7) and acetamide (C9/C10/N2/O3) mean planes is 89.08 (7)°, while between the acetamide (C9/C10/N2/O3) and acetic acid (C11/C12/O4/O5) mean planes the dihedral angle is 83.21 (7)°.

The phthalimide group is planar and the bond lengths and angles are within normal ranges (Ng, 1992; Feeder & Jones, 1996). The acetamide and acetic acid groups have trigonal planar geometry with the sum of the bond angles being 359.98 ° and 359.96 °, respectively. The CN bond lengths in the acetamide moiety, [C10—N2 1.3290 (14) Å and C11—N2 1.4546 (16) Å] are very close to those expected for double and single CN bonds, respectively (Allen *et al.*, 1987). The C=O bond length [C10-O3 = 1.2399 (14) Å] is significantly longer than the C—O bond length in the acetic acid moiety [C12—O4 = 1.2086 (15) Å]. This suggests that some tautomerism of the type OC—NH and HOC=N exists in the acetamide moiety. The carbon oxygen distances in the carboxylate (COO^-^) group show typical double and single bond values [C12—O4 = 1.2086 (15) Å and C12—O5 = 1.3265 (14) Å, respectively].

In the crystal neighbouring and symmetry related molecules are linked via N-H···O and O-H···O hydrogen bonds to form an undulating two-dimensional network (Fig. 2 and Table 1). Together with a number of intermolecular C-H···O contacts (Table 1) these interactions lead to the formation of a three dimensional arrangement.

### Experimental

The title compound was synthesized by the treatment of N-phthaloylglycyl chloride (30 mmol) with potassium thiocyanate (30 mmol) in dry acetone (50 ml). The mixture was stirred at 328 - 333 K for 1 h, followed by the addition of glycine (30 mmol) and a few drops of pyridine, and then refluxed for 6 h. After reflux, the mixture was treated with ice cold water untill a precipitate appeared, which was collected by filtration, washed with water, and recrystallized with ethanol to give colourless block-like crystals, suitable for X-ray diffraction analysis.

### Refinement

The OH and NH H-atoms were located in a difference electron density map and were freely refined: N-H = 0.908 (19) Å, O-H = 0.93 (3) Å. The C-bound H-atoms were included in calculated positions and treated as riding: C-H = 0.95 and 0.99Å for CH and CH_2_ H-atoms, respectively, with U_iso_(H) = 1.2U_eq_(C).

### Crystal data

**Table:**

C_12_H_10_N_2_O_5_	*F*(000) = 544
*M**_r_* = 262.22	*D*_x_ = 1.544 Mg m^−^^3^
Monoclinic, *P*2_1_/*n*	Mo *K*α radiation, λ = 0.71073 Å
Hall symbol: -P 2yn	Cell parameters from 6788 reflections
*a* = 4.8195 (5) Å	θ = 2.7–28.3°
*b* = 10.3415 (11) Å	µ = 0.12 mm^−^^1^
*c* = 22.629 (2) Å	*T* = 173 K
β = 90.17 (1)°	Block, colorless
*V* = 1127.9 (2) Å^3^	0.34 × 0.24 × 0.20 mm
*Z* = 4	

### Data collection

**Table:**

Bruker SMART CCD diffractometer	2731 independent reflections
Radiation source: fine-focus sealed tube	2533 reflections with *I* > 2σ(*I*)
graphite	*R*_int_ = 0.015
ω and φ scans	θ_max_ = 28.3°, θ_min_ = 2.7°
Absorption correction: multi-scan (*SADABS*; Sheldrick, 1996)	*h* = −5→6
*T*_min_ = 0.939, *T*_max_ = 1.000	*k* = −6→13
6788 measured reflections	*l* = −29→29

### Refinement

**Table:**

Refinement on *F*^2^	Primary atom site location: structure-invariant direct methods
Least-squares matrix: full	Secondary atom site location: difference Fourier map
*R*[*F*^2^ > 2σ(*F*^2^)] = 0.037	Hydrogen site location: inferred from neighbouring sites
*wR*(*F*^2^) = 0.094	H atoms treated by a mixture of independent and constrained refinement
*S* = 1.07	*w* = 1/[σ^2^(*F*_o_^2^) + (0.037*P*)^2^ + 0.579*P*] where *P* = (*F*_o_^2^ + 2*F*_c_^2^)/3
2731 reflections	(Δ/σ)_max_ < 0.001
180 parameters	Δρ_max_ = 0.33 e Å^−^^3^
0 restraints	Δρ_min_ = −0.24 e Å^−^^3^

### Special details

**Table:**

Geometry. Bond distances, angles etc. have been calculated using the rounded fractional coordinates. All su's are estimated from the variances of the (full) variance-covariance matrix. The cell esds are taken into account in the estimation of distances, angles and torsion angles
Refinement. Refinement of *F*^2^ against ALL reflections. The weighted *R*-factor *wR* and goodness of fit *S* are based on *F*^2^, conventional *R*-factors *R* are based on *F*, with *F* set to zero for negative *F*^2^. The threshold expression of *F*^2^ > σ(*F*^2^) is used only for calculating *R*-factors(gt) *etc*. and is not relevant to the choice of reflections for refinement. *R*-factors based on *F*^2^ are statistically about twice as large as those based on *F*, and *R*- factors based on ALL data will be even larger.

### Fractional atomic coordinates and isotropic or equivalent isotropic displacement parameters (Å^2^)

**Table:**

	*x*	*y*	*z*	*U*_iso_*/*U*_eq_	
O1	1.42937 (19)	0.17860 (9)	0.07903 (4)	0.0274 (3)	
O2	0.7194 (2)	0.46000 (10)	0.04263 (4)	0.0338 (3)	
O3	1.11056 (19)	0.36795 (9)	0.23395 (4)	0.0250 (3)	
O4	0.8527 (2)	0.07998 (11)	0.31666 (4)	0.0333 (3)	
O5	1.05382 (19)	0.04425 (9)	0.22876 (4)	0.0264 (3)	
N1	1.0744 (2)	0.32888 (10)	0.07534 (4)	0.0205 (3)	
N2	0.8077 (2)	0.25359 (10)	0.18003 (4)	0.0214 (3)	
C1	1.1901 (3)	0.10108 (14)	−0.04587 (6)	0.0285 (4)	
C2	1.0418 (3)	0.09730 (15)	−0.09889 (6)	0.0336 (4)	
C3	0.8300 (3)	0.18476 (15)	−0.11078 (6)	0.0329 (4)	
C4	0.7551 (3)	0.27995 (14)	−0.06977 (6)	0.0279 (4)	
C5	0.9028 (2)	0.28280 (12)	−0.01754 (5)	0.0223 (3)	
C6	1.1163 (2)	0.19590 (12)	−0.00576 (5)	0.0219 (3)	
C7	1.2343 (2)	0.22725 (12)	0.05335 (5)	0.0208 (3)	
C8	0.8738 (2)	0.37080 (12)	0.03419 (5)	0.0223 (3)	
C9	1.1328 (2)	0.39863 (11)	0.12934 (5)	0.0206 (3)	
C10	1.0158 (2)	0.33726 (11)	0.18492 (5)	0.0190 (3)	
C11	0.6866 (2)	0.19718 (13)	0.23289 (6)	0.0245 (3)	
C12	0.8745 (2)	0.10203 (12)	0.26443 (5)	0.0218 (3)	
H1A	1.33480	0.04130	−0.03780	0.0340*	
H2	0.743 (4)	0.2331 (18)	0.1435 (8)	0.037 (5)*	
H2A	1.08690	0.03340	−0.12750	0.0400*	
H3A	0.73430	0.17980	−0.14740	0.0390*	
H4A	0.60970	0.33960	−0.07750	0.0340*	
H5	1.159 (4)	−0.018 (2)	0.2480 (8)	0.043 (5)*	
H9A	1.05660	0.48720	0.12570	0.0250*	
H9B	1.33640	0.40630	0.13380	0.0250*	
H11A	0.51250	0.15240	0.22190	0.0290*	
H11B	0.63790	0.26770	0.26060	0.0290*	

### Atomic displacement parameters (Å^2^)

**Table:**

	*U*^11^	*U*^22^	*U*^33^	*U*^12^	*U*^13^	*U*^23^
O1	0.0269 (4)	0.0280 (5)	0.0271 (5)	0.0052 (4)	−0.0068 (4)	−0.0024 (4)
O2	0.0349 (5)	0.0362 (5)	0.0302 (5)	0.0138 (4)	−0.0064 (4)	−0.0023 (4)
O3	0.0277 (4)	0.0293 (5)	0.0180 (4)	−0.0050 (4)	−0.0041 (3)	0.0004 (3)
O4	0.0375 (5)	0.0401 (6)	0.0224 (4)	0.0032 (4)	0.0029 (4)	0.0082 (4)
O5	0.0292 (5)	0.0281 (5)	0.0218 (4)	0.0061 (4)	−0.0019 (3)	0.0023 (4)
N1	0.0219 (5)	0.0227 (5)	0.0168 (4)	0.0014 (4)	−0.0018 (4)	−0.0003 (4)
N2	0.0225 (5)	0.0223 (5)	0.0195 (5)	−0.0013 (4)	−0.0043 (4)	0.0029 (4)
C1	0.0313 (6)	0.0291 (7)	0.0251 (6)	−0.0009 (5)	0.0007 (5)	−0.0051 (5)
C2	0.0411 (7)	0.0366 (7)	0.0232 (6)	−0.0061 (6)	0.0011 (5)	−0.0094 (5)
C3	0.0370 (7)	0.0434 (8)	0.0183 (6)	−0.0096 (6)	−0.0047 (5)	−0.0007 (5)
C4	0.0281 (6)	0.0352 (7)	0.0205 (6)	−0.0041 (5)	−0.0039 (5)	0.0045 (5)
C5	0.0235 (5)	0.0254 (6)	0.0181 (5)	−0.0028 (5)	0.0002 (4)	0.0020 (4)
C6	0.0227 (5)	0.0243 (6)	0.0187 (5)	−0.0036 (4)	−0.0009 (4)	0.0002 (4)
C7	0.0219 (5)	0.0209 (5)	0.0197 (5)	−0.0021 (4)	0.0001 (4)	−0.0003 (4)
C8	0.0225 (5)	0.0260 (6)	0.0185 (5)	0.0000 (4)	−0.0014 (4)	0.0033 (4)
C9	0.0235 (5)	0.0210 (5)	0.0173 (5)	−0.0016 (4)	−0.0013 (4)	−0.0008 (4)
C10	0.0201 (5)	0.0186 (5)	0.0182 (5)	0.0028 (4)	−0.0020 (4)	0.0003 (4)
C11	0.0200 (5)	0.0275 (6)	0.0259 (6)	−0.0005 (5)	0.0012 (4)	0.0054 (5)
C12	0.0207 (5)	0.0219 (6)	0.0228 (6)	−0.0051 (4)	−0.0021 (4)	0.0021 (4)

### Geometric parameters (Å, °)

**Table:**

O1—C7	1.2130 (14)	C3—C4	1.401 (2)
O2—C8	1.2008 (15)	C4—C5	1.3782 (18)
O3—C10	1.2399 (14)	C5—C8	1.4896 (17)
O4—C12	1.2086 (15)	C5—C6	1.3913 (16)
O5—C12	1.3265 (14)	C6—C7	1.4877 (16)
O5—H5	0.93 (2)	C9—C10	1.5188 (16)
N1—C8	1.4087 (14)	C11—C12	1.5146 (17)
N1—C9	1.4459 (15)	C1—H1A	0.9500
N1—C7	1.3959 (15)	C2—H2A	0.9500
N2—C10	1.3290 (14)	C3—H3A	0.9500
N2—C11	1.4546 (16)	C4—H4A	0.9500
N2—H2	0.908 (18)	C9—H9A	0.9900
C1—C2	1.395 (2)	C9—H9B	0.9900
C1—C6	1.3834 (18)	C11—H11A	0.9900
C2—C3	1.390 (2)	C11—H11B	0.9900
			
C12—O5—H5	112.5 (11)	O3—C10—N2	121.14 (10)
C7—N1—C9	124.79 (9)	O3—C10—C9	119.83 (10)
C8—N1—C9	122.45 (10)	N2—C11—C12	114.03 (9)
C7—N1—C8	112.00 (9)	O4—C12—O5	124.66 (11)
C10—N2—C11	119.81 (10)	O4—C12—C11	121.99 (11)
C10—N2—H2	119.0 (12)	O5—C12—C11	113.31 (10)
C11—N2—H2	121.2 (12)	C2—C1—H1A	121.00
C2—C1—C6	116.87 (13)	C6—C1—H1A	122.00
C1—C2—C3	121.49 (13)	C1—C2—H2A	119.00
C2—C3—C4	121.31 (13)	C3—C2—H2A	119.00
C3—C4—C5	116.72 (13)	C2—C3—H3A	119.00
C4—C5—C8	129.61 (11)	C4—C3—H3A	119.00
C4—C5—C6	122.03 (11)	C3—C4—H4A	122.00
C6—C5—C8	108.37 (9)	C5—C4—H4A	122.00
C1—C6—C5	121.57 (11)	N1—C9—H9A	109.00
C1—C6—C7	130.24 (11)	N1—C9—H9B	108.00
C5—C6—C7	108.18 (10)	C10—C9—H9A	108.00
N1—C7—C6	105.94 (9)	C10—C9—H9B	109.00
O1—C7—C6	129.35 (11)	H9A—C9—H9B	108.00
O1—C7—N1	124.71 (11)	N2—C11—H11A	109.00
O2—C8—N1	123.76 (11)	N2—C11—H11B	109.00
O2—C8—C5	130.84 (10)	C12—C11—H11A	109.00
N1—C8—C5	105.41 (9)	C12—C11—H11B	109.00
N1—C9—C10	114.81 (9)	H11A—C11—H11B	108.00
N2—C10—C9	119.02 (10)		
			
C9—N1—C7—C6	−173.65 (10)	C3—C4—C5—C8	−179.94 (13)
C7—N1—C8—O2	−177.27 (11)	C3—C4—C5—C6	0.06 (19)
C9—N1—C8—O2	−6.78 (17)	C4—C5—C6—C1	−0.53 (19)
C7—N1—C8—C5	2.75 (12)	C6—C5—C8—N1	−0.90 (12)
C8—N1—C7—O1	176.11 (11)	C8—C5—C6—C7	−1.12 (12)
C9—N1—C7—O1	5.88 (18)	C4—C5—C6—C7	178.88 (11)
C8—N1—C7—C6	−3.42 (12)	C8—C5—C6—C1	179.47 (11)
C8—N1—C9—C10	104.76 (12)	C6—C5—C8—O2	179.13 (12)
C9—N1—C8—C5	173.24 (9)	C4—C5—C8—O2	−0.9 (2)
C7—N1—C9—C10	−85.99 (12)	C4—C5—C8—N1	179.11 (12)
C11—N2—C10—O3	0.39 (17)	C1—C6—C7—O1	2.6 (2)
C11—N2—C10—C9	−177.90 (10)	C5—C6—C7—O1	−176.76 (12)
C10—N2—C11—C12	−70.18 (14)	C5—C6—C7—N1	2.74 (12)
C2—C1—C6—C7	−178.78 (12)	C1—C6—C7—N1	−177.93 (12)
C2—C1—C6—C5	0.48 (19)	N1—C9—C10—O3	161.21 (10)
C6—C1—C2—C3	0.0 (2)	N1—C9—C10—N2	−20.48 (14)
C1—C2—C3—C4	−0.5 (2)	N2—C11—C12—O4	154.71 (12)
C2—C3—C4—C5	0.4 (2)	N2—C11—C12—O5	−27.42 (14)

### Hydrogen-bond geometry (Å, °)

**Table:**

*D*—H···*A*	*D*—H	H···*A*	*D*···*A*	*D*—H···*A*
N2—H2···O1^i^	0.908 (18)	2.172 (19)	3.0208 (13)	155.3 (17)
O5—H5···O3^ii^	0.93 (2)	1.67 (2)	2.5777 (13)	165.4 (17)
C2—H2A···O5^iii^	0.95	2.52	3.3142 (17)	141
C9—H9A···O4^iv^	0.99	2.56	3.2407 (15)	126
C9—H9B···O4^v^	0.99	2.59	3.3378 (15)	132
C11—H11A···O5^i^	0.99	2.48	3.4364 (14)	162

Symmetry codes: (i) *x*−1, *y*, *z*; (ii) −*x*+5/2, *y*−1/2, −*z*+1/2; (iii) −*x*+2, −*y*, −*z*; (iv) −*x*+3/2, *y*+1/2, −*z*+1/2; (v) −*x*+5/2, *y*+1/2, −*z*+1/2.
